# The impact of albumin infusion on the risk of rebleeding and in-hospital mortality in cirrhotic patients admitted for acute gastrointestinal bleeding: a retrospective study of a single institute

**DOI:** 10.1186/s12876-020-01337-5

**Published:** 2020-06-23

**Authors:** Zhu Wang, Ya-Wen Xie, Qing Lu, Hai-Lin Yan, Xin-Bin Liu, Yi Long, Xian Zhang, Jin-Lin Yang

**Affiliations:** 1 Department of Gastroenterology and Hepatology, West China Hospital of Sichuan University, 37# Guoxue Lane, Chengdu, 610041 Sichuan China; 2 West China School of Medicine of Sichuan University, Chengdu, China

**Keywords:** Albumin, Cirrhosis, Gastrointestinal bleeding, Portal hypertension

## Abstract

**Background:**

To investigate the effect of albumin infusion on cirrhotic patients admitted for acute gastrointestinal bleeding.

**Methods:**

Medical records of cirrhotic patients who admitted due to acute gastrointestinal bleeding through January 2009 to December 2018 were reviewed. Clinical data and the total amount of albumin and red blood cell used during hospitalization were recorded. For patients with rebleeding, the amount of albumin and red blood cell used before rebleeding was also documented. The primary outcome was the occurrence of rebleeding, and the second outcome was in-hospital mortality. Univariate and multivariate logistic analysis was performed to identify risk factors associated with rebleeding and in-hospital mortality.

**Results:**

A total of 1503 cirrhotic patients were included in the analysis. There were 146 episodes of in-patient rebleeding occurred, while 81 patients died. Overall, more red blood cells and albumin were prescribed to patients who suffered rebleeding. In terms of the amount before rebleeding, the red blood cell was higher in patients with rebleeding, but the albumin infusion was similar. In the multivariate model, the albumin infusion before rebleeding was an independent risk factor associated with rebleeding (adjusted OR for ≤40 g vs 0 g, 0.469 [0.269–0.793], *p* = 0.006; adjusted OR for > 40 g vs 0 g, 0.272 [0.115–0.576], *p* = 0.001). In Child-Pugh C class patients, the use of albumin more than 40 g during hospitalization associated with a lower risk of in-patient mortality (adjusted OR for > 40 g vs 0 g, 0.136 [0.019–0.741], *p* = 0.031).

**Conclusions:**

Albumin infusion was associated with a lower risk of rebleeding and in-hospital deaths in cirrhosis admitted for acute gastrointestinal bleeding.

## Background

Gastrointestinal bleeding (GIB) accounts for nearly one-third of all death in cirrhotic patients. Hypoalbuminemia, frequently caused by the impaired synthesis in the scenario of advanced cirrhosis, may further deteriorate after GIB due to the direct loss of albumin in the gastrointestinal tract and short-term fast. As an established risk factor for sepsis and mortality in critically ill patients, hypoalbuminemia needs to be corrected by albumin treatment [1]. In patients with cirrhosis, the use of albumin prevents circulatory dysfunction after paracentesis, improves the outcomes in patients with refractory ascites, spontaneous bacterial peritonitis (SBP) and hepatorenal syndrome (HRS) [2, 3]. However, no studies thus far have elucidated its role in the management of GIB in cirrhotic patients.

The antibiotic prophylaxis has been accepted by international consensus for its role in reducing rebleeding risk in cirrhotic patients [4]. The therapeutic effect of antibiotics in cirrhosis is attributed to the improvement of the inflammatory state caused by bacterial infection [5]. Meanwhile, emerging evidence supports the anti-inflammatory property of albumin, which contributes to reducing the risk of complications and mortality rate in cirrhotic patients [6, 7]. On the contrary, albumin infusion, noted as a volume expander, may increase portal pressure and induce rebleeding due to deteriorated pre-existing portal hypertension [8]. Therefore, our study aims to investigate whether the albumin infusion would affect the prognosis of cirrhotic patients admitted for GIB.

## Methods

### Study design

Medical records of cirrhotic patients who admitted to West China Hospital due to GIB through Jan 2009 to Dec 2018 were systemically reviewed. Data of patients’ demographics, cause of cirrhosis, Child-Pugh classification, laboratory tests, days of hospital stay, sources of bleeding, initial rescue therapy with balloon tamponade, endoscopic and radiological interventions, the concomitant of hepatic carcinoma (HCC) and portal vein thrombosis (PVT), the occurrence of in-hospital rebleeding and deaths were collected based on the medical records.

The sources of bleeding were defined as the upper or lower GI tract according to endoscopic findings. The upper GI bleeding was further divided into variceal and non-variceal bleeding lesions as determined by esophagogastroduodenoscopy. Variceal bleeding was defined as active hemorrhage from varices, or the presence of a clot over varices, or varices as the only potential source of bleeding. Patients with multiple suspected bleeding lesions were recorded respectively. The presence of HCC or PVT was confirmed by radiological imaging records or discharge diagnosis. The baseline values of hemoglobin, bilirubin, albumin, creatine, and prothrombin time were obtained from the laboratory results at admission. Patients with HCC or PVT were excluded from further analysis.

### Clinical outcomes

The primary outcome was the in-hospital rebleeding event, defined as the following symptoms documented in the medical records that occurred after initial hemostasis achieved: 1) new occurrence of hematemesis or bloody nasogastric aspirate; 2) recurrence of hematochezia or melena. The secondary outcome was in-hospital mortality. The death reasons were derived from the discharge diagnosis.

### Infusion of albumin and red blood cell

The amount of albumin and red blood cell (RBC) infusion during hospitalization was collected from the hospital database. The total amount of albumin and RBC used during hospitalization were recorded as total-ALB and total-RBC. For patients with rebleeding occurred, the amount of albumin and RBC used before rebleeding was recorded as pre-ALB and pre-RBC. For patients without rebleeding, the amount of pre-ALB and pre-RBC was equivalent to the total-ALB and total-RBC.

### Statistical analysis

Continuous variables were displayed as means ± standard deviation and compared using the Student’s *t* test. Categorical variables were displayed as frequencies and percentages and compared by Chi-square or Fisher exact tests. Baseline characteristics and other associated covariates with outcomes were estimated with univariate and multivariate logistic regression models and reported as odds ratios (OR) with 95% confidence intervals (CI). Statistics are calculated using R version 3.2.0 (R Foundation for Statistical Computing, Vienna, Austria).

## Results

### Patients’ baseline characteristics and outcomes

A total of 2259 records of cirrhotic patients admitted for acute GIB were selected, among whom 20 cases were excluded for the incomplete data. There were 736 patients with PVT or HCC were excluded. Overall, 1503 records were included in this study. The mean age was 53.1 ± 13.1, 1005 (66.9%) were male. Except for 190 (12.6%) cases without identified etiology, the other etiologies for cirrhosis were: 867 (57.7%) HBV infections; 197 (13.1%) alcoholic liver diseases; 135 (9.0%) autoimmune liver diseases; 59 (3.9%) HCV infections; 22 (1.5%) secondary cholestatic liver diseases; 10 (0.7%) vascular disorders, 23 (1.5%) others. According to the Child-Pugh classification, there were 474 (31.5%) patients classified as class A, 715 (47.6%) as class B, and 314 (20.9%) as class C. At admission, 123 patients were concomitant with encephalopathy, and 750 patients had ascites. In terms of the rebleeding sources, there were 128 (8.5%) patients who did not receive endoscopy examinations to identify the bleeding lesion (unidentified lesion). Because they could not tolerate interventions due to comorbidities or patients avoid endoscopic interventions. There were 1375 (91.5%) cases presented upper GI tract bleeding lesion (variceal or non-variceal lesions) and 47 (3.1%) had lower GI tract bleeding lesions (jejunoileal or colonic lesion). Variceal lesion were observed in 1275 cases (84.8%), while non-variceal lesion in 100 patients (6.7%). In variceal bleeding, balloon tamponade was initially applied in 169 cases to control active bleeding, 21 emergent transjugular intrahepatic portosystemic shunts (TIPS) were performed as rescue therapy. During hospitalization, 511 (34.0%) patients received endoscopic treatments, including 491 cases of endoscopic band ligation with/without injection of tissue adhesive, 19 cases of endoscopic sclerotherapy/injection of tissue adhesive, 1 cases endoscopic treatment for ulcer lesions. About half of the endoscopic treatments (251, 49.1%) were performed within 5 days to prevent early rebleeding. There were 10 cases underwent additional radiological interventional treatment due to the failure of endoscopic treatments, while 372 (25.0%) patients only underwent radiological interventional treatment. The majority of the radiological interventions were TIPS (352, 92.1%) for the failure of previous endoscopic treatment prevention. The other interventions including balloon-occluded retrograde obliteration (20, 5.2%), embolization of non-variceal lesion (8, 2.1%), and partial splenic embolization (2, 0.5%).

Overall, there were 146 (9.7%) patients for whom in-patient rebleeding occurred, with an average of 12.2 ± 6.5 hospitalization days. As regards the in-hospital mortality, a total of 81 (5.4%) patients died during hospitalization and 67 of them experienced in-patient rebleeding before death. The reasons of deaths were as followed: hemorrhagic shock (*n* = 57), hepatorenal syndrome (*n* = 11), hepatic encephalopathy (*n* = 1), multiple organ failure (*n* = 6), infection (*n* = 2), cerebrovascular event (*n* = 3), and acute myocardial infarction (*n* = 1).

### The association between albumin/RBC infusion for in-patient rebleeding

Univariate variables associated with rebleeding were displayed in Table 1. Patients suffered rebleeding were more likely to have hepatic encephalopahty (6.9% vs 19.9%, *p* < 0.001), higher Child-Pugh classification (Child A 33.5% vs 13.0%, Child B 47.1% vs 52.1%, Child C 19.4% vs 34.9%, *p* < 0.001), higher bilirubin level (32.5 vs 48.4 umol/L, *p* < 0.001), lower albumin level (31.0 vs 27.8 g/L, *p* < 0.001), and deteriorated prothrombin time at baseline (16.6 vs 18.3 s, *p* = 0.038). More rebleeding occurred in cases with unidentified lesions (8.0% vs 13.7%, *p* = 0.027) and patients without ascites (51.5% vs 34.9%, *p* < 0.001). Rebleeding was more likely to occur in patients rescued by balloon tamponade at admission (7.0% vs 50.7%, *p* < 0.001), while subsequent endoscopic treatment effectively prevents rebleeding (36.0% vs 15.1%, *p* < 0.001) (Table 1).

**Table 1:** Univariate analysis of baseline characteristics between patients with and without rebleeding

	No rebleeding (*n* = 1357)	Rebleeding (*n* = 146)	*P* value
Age	52.9 ± 13.0	54.6 ± 13.3	0.144
Gender (Male)	907 (66.8)	98 (67.1)	1
Child-Pugh class			< 0.001
A	455 (33.5)	19 (13.0)	
B	639 (47.1)	76 (52.1)	
C	263 (19.4)	51 (34.9)	
Hepatic encephalopathy	94 (6.9)	29 (19.9)	< 0.001
Ascites	699 (51.5)	51 (34.9)	< 0.001
Source of Bleeding			
Varices lesion	1154 (85.0)	121 (82.9)	0.568
Non-varices lesion	96 (7.1)	4 (2.7)	0.068
Lower gastrointestinal lesion	43 (3.2)	4 (2.7)	0.974
Unidentified lesion	108 (8.0)	20 (13.7)	0.027
Bilirubin (umol/L) ^a^	32.5 ± 40.6	48.4 ± 88.4	< 0.001
Albumin (g/L) ^a^	31.0 ± 6.1	27.8 ± 6.6	< 0.001
Creatine (mol/L) ^a^	80.6 ± 60.3	90.1 ± 58.5	0.071
Hemoglobin (g/L) ^a^	75.8 ± 22.9	73.7 ± 22.3	0.274
Prothrombin time (s) ^a^	16.6 ± 9.7	18.3 ± 10.5	0.038
Balloon tamponade	95 (7.0)	74 (50.7)	< 0.001
Endoscopic treatment	489 (36.0)	22 (15.1)	< 0.001
Radiological intervention	336 (24.8)	46 (31.5)	0.093

^a^values were obtained at admission

An average of 2.3 ± 3.3 units of RBC and 14.2 ± 32.3 g albumin were administrated to patients presented anemia or hypoalbuminemia. During hospitalization, the average minimum value of HGB and albumin were significantly higher in those patients without rebleeding (Table 2). Hence, more total-RBC (1.9 ± 2.5 vs 6.7 ± 5.9 units, *p* < 0.001) and total-ALB (12.7 ± 28.7 vs 28.5 ± 53.3 g, *p* < 0.001) were prescribed to patients who had rebleeding, compared to those without rebleeding. Nevertheless, the pre-RBC transfusion was higher in rebleeding patients (1.9 ± 2.5 vs 4.1 ± 3.9 units, *p* < 0.001), while the pre-ALB infusion was comparable (12.7 ± 28.7 vs 10.5 ± 34.4 g, *p* = 0.395). In particular, in patients with Child-Pugh C class, the pre-ALB infusion was lower in those with rebleeding (14.1 vs 29.9 g, *p* = 0.021). (Table 2).

**Table 2:** The amount of albumin and RBC infusion in cirrhosis with and without in-patient rebleeding

	Child-Pugh A			Child-Pugh B			Child-Pugh C		
	No rebleeding	rebleeding	*P* value	No rebleeding	rebleeding	*P* value	No rebleeding	rebleeding	*P* value
Albumin (g/L)^b^	32.9	24.6	< 0.001	27.9	23	< 0.001	24.3	20.9	0.001
Total-ALB (g) ^a^	3.0	18.3	< 0.001	12.5	30.3	< 0.001	29.9	29.7	0.974
Pre-ALB (g) ^c^	3.0	2.2	0.790	12.5	10.2	0.462	29.9	14.1	0.021
HGB (g/L) ^b^	71.9	57.2	< 0.001	65.6	52.6	< 0.001	61.7	55.2	0.041
Total-RBC (unit) ^a^	1.2	6.0	< 0.001	2.0	7.5	< 0.001	2.5	5.8	< 0.001
Pre-RBC (unit) ^c^	1.2	3.1	< 0.001	2.0	4.8	< 0.001	2.5	3.5	0.032

^a^Total-ALB and total-RBC: the total amount of albumin/RBC infusion during hospitalization;

^b^The average minimum value of albumin or hemoglobin during hospitalization;

^c^Pre-ALB and pre-RBC: the amount of albumin infusion before rebleeding occurrence. In patients without bleeding, the pre-ALB and pre-RBC equals the total-ALB and total-RBC

To explore the association between the dose and risk of rebleeding, the amount of pre-ALB was further classified into two subgroups as ≤40 g and >  40 g. The use of pre-ALB was not linked to rebleeding in univariate analysis. In the multivariate model adjusted for baseline albumin and Child-Pugh class, however, the use of an increased dose of albumin reduces the risk of rebleeding (OR for ≤40 g vs 0 g, 0.500 [0.312–0.800], *p* = 0.004; OR for > 40 g vs 0 g, OR 0.279 [0.134–0.580], *p* < 0.001).(Table 3).

**Table 3:** Multivariate Hazards regression analysis of the relationship between different dose of albumin/red blood cell infusion before rebleeding and rebleeding

Pre-ALB infusion				
	(0, 40 g]	*P* value	> 40 g	*P* value
Unadjusted	0.879 (0.561–1.376)	0.572	0.575 (0.285–1.160)	0.122
Adjusted for albumin, Child-Pugh class	0.500 (0.312–0.800)	0.004	0.279 (0.134–0.580)	< 0.001
Multivariable ^a^adjusted	0.469 (0.269-0.793)	0.006	0.272 (0.115-0.576)	0.001
Pre-RBC infusion				
	(4–8 units]	*P* value	> 8 units	*P* value
Unadjusted	3.556 (2.369–5.338)	< 0.001	5.654 (2.913–10.975)	< 0.001
Adjusted for hemoglobin, Child-Pugh class	3.497 (2.229–5.485)	< 0.001	5.709 (2.852–11.426)	< 0.001
Multivariable ^a^ adjusted	1.888 (1.144-3.067)	0.011	2.634 (1.142-5.812)	0.019

^a^adjust for Child-Pugh classification, presence of encephalopathy and ascties, bleed from unidentified lesion, the initial application of tamponade, values of bilirubin, albumin and prothrombin time, endoscopic treatments

**Fig. 1:**
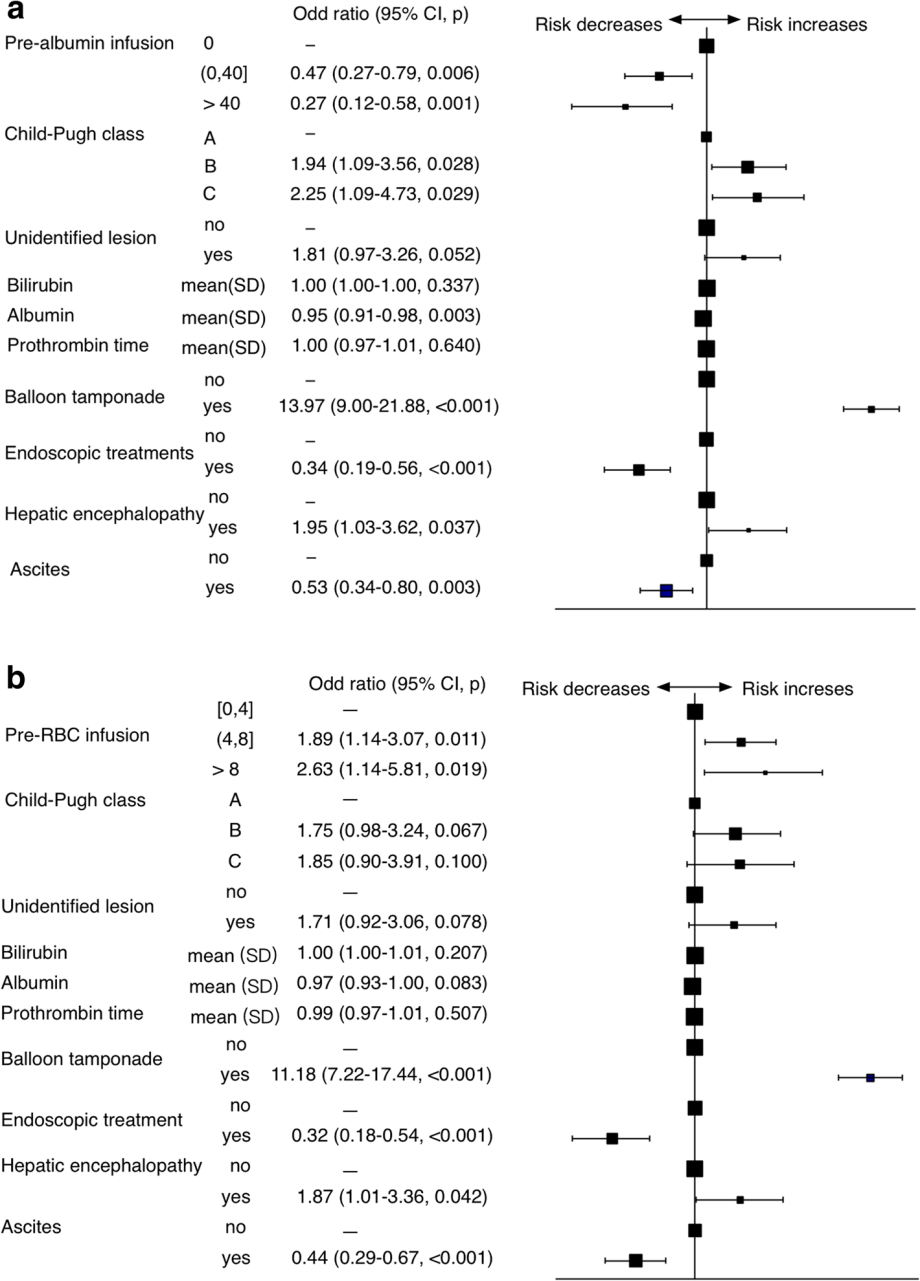
Multivariate hazard regression of (**a**) different dose (g) of albumin infusion before rebleeding (pre-ALB); (**b**) different dose (units) of red blood cell infusion (pre-RBC) and rebleeding event

Furthermore, in another multivariate model adjusted for all confounders (Child-Pugh class, presence of hepatic encephalopathy and ascites, bleed from unidentified lesion, the initial application of balloon tamponade, values of bilirubin, albumin and prothrombin time, endoscopic treatments), the pre-ALB infusion was still associated with a lower risk of rebleeding. There was a negative dose-effect relationship between albumin and rebleeding risk (adjusted OR for ≤40 g vs 0 g, 0.469 [0.269-0.793], *p* = 0.006; adjusted OR for > 40 g vs 0 g, 0.272 [0.115-0.576], *p* = 0.001). Other independent factors in this model included Child-Pugh classification (adjusted OR for Child B vs A, 1.935 [1.090–3.563], *p* = 0.028; Child C vs A, 2.253 [1.094–4.730], *p* = 0.029), presence of encephalopathy (adjusted OR 1.951 [1.029–3.621], *p* = 0.037) and ascites (adjusted OR 0.526 [0.344–0.799], *p* = 0.003), rescue therapy with balloon tamponade (adjusted OR 13.996 [9.002-21.882], *p* < 0.001) and endoscopic treatment (adjusted OR 0.337 [0.195–0.561], *p* < 0.001) (Fig. 1a).

Regarding RBC infusion before rebleeding, the pre-RBC infusion remained associated with more occurrences of rebleeding in univariate (OR for 4–8 vs < 4 units 3.556 [2.369–5.338], *p* < 0.001; OR for > 8 vs < 4 units 5.654 [2.913–10.975], *p* < 0.001). In the multivariate analysis adjusted for the confounders above, the risk was positively correlated with the infused RBC units (adjusted OR for 4–8 vs < 4 units 1.888 [1.144-3.067], *p* = 0.011; adjusted OR for > 8 vs < 4 units 2.634 [1.142-5.812], *p* = 0.019) (Fig. 1b).

When the multivariate analysis were further performed stratified by Child-Pugh class, the benefit effect of pre-ALB infusion was only observed in patients of Child-Pugh C class (adjusted OR for ≤40 g vs 0 g, 0.185 [0.057–0.520], *p* = 0.002; adjusted OR for > 40 g vs 0 g, 0.198 [0.047–0.668], *p* = 0.015). While, the negative impact of pre-RBC infusion was predominantly shown in patients of Child-Pugh B class (adjusted OR for 4–8 vs < 4 units 2.235 [1.168–4.180], *p* = 0.013; adjusted OR for > 8 vs < 4 units 3.495 [1.254–9.373], *p* = 0.014).

### The association between albumin/RBC infusion and in-hospital mortality

**Fig. 2:**
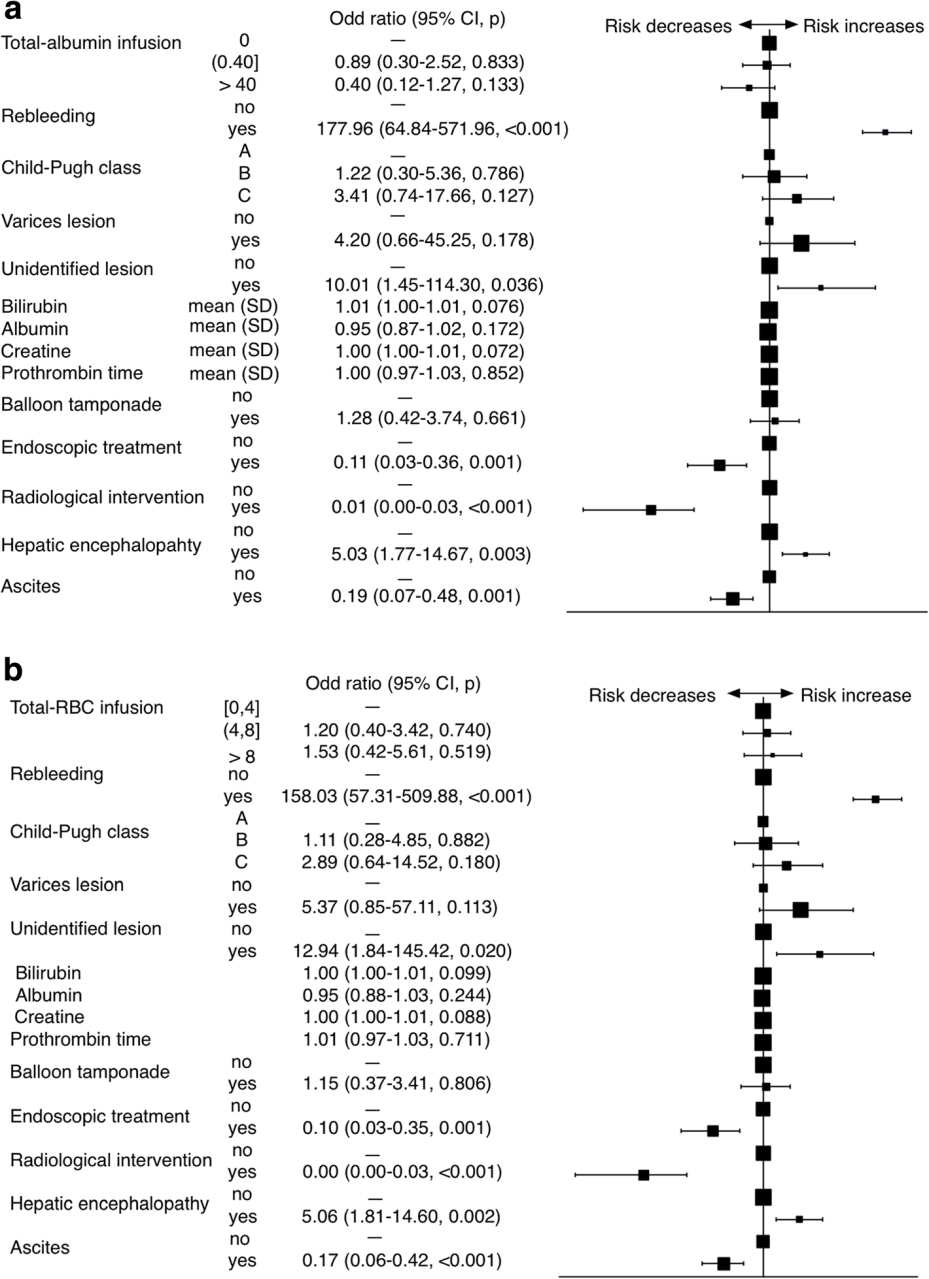
Multivariate hazard regression of (**a**) different dose (g) of albumin infusion before rebleeding (total-ALB); (**b**) different dose (units) of red blood cell (total-RBC) infusion and mortality

In the univariate analysis, the in-hospital mortality was more probable in patients with compromised liver function (i.e. higher Child-Pugh classification, lower values of albumin, higher values of bilirubin, creatine and prothrombin time), prensece of encephalopathy, bleed from varices or unidentified lesion, initial rescue therapy with tamponade and occurrence of rebleeding. The presence of ascites, the uses of endoscopic and radiological interventions decreased mortality risk (Table 4). A higher amount of total-RBC (2.2 ± 3.1 vs 5.5 ± 5.3 units, *p* < 0.001) and similary amount of total-ALB (13.9 ± 31.0 g vs 19.8 ± 49.1 g, *p* = 0.114) infused to patients who died during hospitalization. However, after adjusted for baseline albumin level and Child-Pugh class, there was a significant correlation between total-ALB infusion and mortality (OR for ≤40 g vs 0 g, 0.485 [0.265–0.888], p = 0.019; OR for > 40 g vs 0 g, 0.432 [0.206–0.903], p = 0.026). In another multivariate model adjusted for all confounders (Child-Pugh classification, presence of encephalopathy and ascites, the occurrence of rebleeding, bleed from variceal lesion or unidentified lesion, the initial therapy of tamponade, values of bilirubin, creatine, albumin and prothrombin time, endoscopic and radiological interventions), the infusion of albumin does not correlated with in-hospital mortality. (Table 5 and Fig. 2a).

**Table 4:** Univariate analysis of baseline characteristics and mortality

	No death (*n* = 1422)	Death (*n* = 81)	*P* value
Age	53.0 ± 13.0	54.2 ± 13.5	0.444
Gender (M)	947 (66.6)	58 (71.6)	0.418
Child-Pugh classification			< 0.001
A	469 (33.0)	5 (6.2)	
B	689 (48.5)	26 (32.1)	
C	264 (18.6)	50 (61.7)	
Hepatic encephalopathy	94 (6.6)	29 (35.8)	< 0.001
Ascites	738 (51.9)	12 (14.8)	< 0.001
Rebleeding	79 (5.6)	67 (82.7)	< 0.001
Sources of bleeding			
Varies lesion	1215 (85.4)	60 (74.1)	0.009
Non-varices lesion	99 (7.0)	1 (1.2)	0.075
Lower gastrointestinal lesion	45 (3.2)	2 (2.5)	0.983
Unidentified lesion	109 (7.7)	19 (23.5)	< 0.001
Bilirubin (umol /L) ^a^	31.8 ± 39.0	74.1 ± 116.7	< 0.001
Albumin (g/L) ^a^	30.9 ± 6.1	26.6 ± 6.5	< 0.001
Creatine (mol/L) ^a^	79.7 ± 58.4	114.0 ± 80.0	< 0.001
Hemoglobin (g/L) ^a^	75.7 ± 22.8	74.6 ± 24.0	0.683
Prothrombin time (s) ^a^	16.5 ± 9.5	21.0 ± 13.4	< 0.001
Balloon tamponade	133 (9.4)	36 (44.4)	< 0.001
Endoscopic treatment	507 (35.7)	4 (4.9)	< 0.001
Radiological intervention	381 (26.8)	1 (1.2)	< 0.001

^a^values were obtained at admission

**Table 5:** Multivariate Hazards regression analysis of different dose of albumin/red blood cell and death

Total-ALB infusion				
	(0, 40 g]	*P* value	> 40 g	*P* value
Unadjusted	1.068 (0.602–1.894)	0.822	1.160 (0.579–2.323)	0.676
Adjusted for albumin, Child-Pugh class	0.485 (0.265–0.888)	0.019	0.432 (0.206–0.903)	0.026
Multivariable ^a^adjusted	0.892 (0.299-2.522)	0.833	0.403 (0.117-1.268)	0.133
Total-RBC infusion				
	(4, 8 units]	*P* value	> 8 units	*P* value
Unadjusted	2.583 (1.448–4.608)	0.001	8.350 (4.550–15.325)	< 0.001
Adjusted for hemoglobin, Child-Pugh class	2.373 (1.253–4.493)	0.008	8.399 (4.256–16.577)	< 0.001
Multivariable ^a^adjusted	1.198 (0.401-3.418)	0.740	1.526 (0.422–5.608)	0.519

^a^adjust for Child-Pugh classification, presence of encephalopathy and ascites, occurrence of rebleeding, bleed from varices lesion and unidentified lesion, the initial application of tamponade, values of bilirubin, creatine, albumin and prothrombin time, endoscopic and radiological treatment

On the other hand, the infusion of total-RBC increased the mortality risk in the univariate analysis (OR for 4–8 vs <4 units, 2.583 [1.448–4.608, *p* = 0.001; OR for > 8 vs <4 units, 8.350 [4.550–15.325], *p* < 0.001) and a multivariate model adjusted for baseline hemoglobin level and Child-Pugh class (OR for 4–8 vs <4 units, 2.373 [1.253–4.493], *p* = 0.008; OR for > 8 vs <4 units, 8.399 [4.256–16.577], *p* < 0.001). However, this association between RBC use and in-hospital mortality risk was not observed in another multivariate model adjusted for all confounders described above (adjusted OR for 4–8 vs <4 units, 1.198 [0.401-3.418], *p* = 0.740; adjusted OR for > 8 vs <4 units, 1.526 [0.422–5.608], *p* = 0.519) (Fig. 2b).

Similarly, the multivariate analysis was performed in each Child-Pugh Class, the beneficial effect of total-ALB infusion was only observed in patients of C class (adjusted OR for ≤40 g vs 0 g, 0.653 [0.111–3.404], *p* = 0.618; adjusted OR for > 40 g vs 0 g, 0.136 [0.019–0.741], *p* = 0.031). Meanwhile, the total-RBC infusion was still not correlated with in-hospital mortality.

## Discussion

Gastrointestinal bleeding (GIB) is a fatal complication of cirrhosis, especially for those with rupture of varices. The application of endoscopic and radiological interventions has greatly improved the prognosis of patients with acute GIB [9]. In addition to the advances of invasive treatments, the conservative treatments remain an integral part of the management of this medical emergency and are increasingly standardized. The therapeutic value of prophylactic antibiotics and restrict blood transfusion strategy has recently been proved by several clinical studies [5, 8]. However, the role of albumin in the management of GIB in patients with decompensated cirrhosis has not yet been assessed. This study demonstrated that albumin infusion was associated with a lower risk of rebleeding and in-hospital mortality.

Our study revealed a rebleeding rate of 9.7% and overall mortality of 5.4%, which were lower than those described in previous studies possibly due to a shorter follow-up period in our study [10, 11]. Despite the risk factors that are already known for rebleeding, our study found that the unidentified lesion also increased the risk of rebleeding and in-hospital mortality in cirrhotic patients [5, 11]. UK consensus recommended endoscopic interventions to unstable patients with severe acute upper GIB immediately after resuscitation, and to all upper GIB patients within 24 h [12]. However, there was no recommendation about the time interval between bleeding and endoscopic treatment in the 2008 Chinese consensus, for not every hospital had the technical expertise required for these treatments [13]. In the past, treatments for variceal bleeding were usually delivered after hemodynamic hemostasis in our institute. Nevertheless, we tried to offer appropriate endoscopic therapies for variceal lesion within 5 days as to prevent early rebleeding. In recent years, we make efforts to provide timely endoscopic interventions as suggested to improve the prognosis [14].

Our study aims to explore whether albumin infusion influences the outcomes of cirrhotic patients admitted for GIB. Theoretically, abundant albumin infusion in patients with GIB may cause rebleeding by increased portal pressure in a similar manner of liberal transfusion strategy [15]. Interestingly, we found that albumin infusion associated with a lower risk of in-hospital rebleeding. Particularly, this beneficial effect was predominately observed in patients with Child-Pugh C class, who had a higher portal pressure. Also, the dose-dependent effect of albumin in reducing rebleeding does not support the view that a higher dose leads to more bleeding events. In fact, a recent study suggests that the portal pressure was not affected after the infusion of albumin (1.5 g/kg, every week) [6]. Hence, the concern that albumin infusion increases the risk of rebleeding due to deterioration of portal pressure seems unnecessary. On the other hand, albumin is used in cirrhosis for the treatment of SBP and was widely discussed in the sepsis treatment for its immunomodulatory and anti-inflammatory properties [16, 17]. As bacterial infection was identified as a critical determinant of rebleeding in cirrhosis by increasing portal pressure through vasoactive substances [5], the effect of albumin on systemic inflammation provides a potential theoretical basis for its role in reducing bleeding. Moreover, albumin may also control the bleeding risk by promoting the transportation of drugs such as proton pump inhibitor and antibiotics, two essential agents in the management of acute GIB. Indeed, a recent clinical study found that the combination of albumin and antibiotics is superior to antibiotics alone in the control of inflammation [6]. Collectively, the mechanisms involved the benefit of albumin is attributed to its effect on systemic inflammation and pharmacokinetic process, although its other properties may also play a role [18, 19].

The association between albumin infusion and in-hospital mortality was also evaluated. Infection, HRS, and liver failure are the main non-bleeding cause of death in cirrhotic patients with acute GIB [10]. It is reasonable to assume that albumin treatment might ameliorate the number of deaths caused by infection or infection-related organ failures. Although the results showed that more than 40 g of albumin infusion lower risk of mortality in Child C class, we failed to demonstrate the beneficial results of its effect on non-bleeding deaths since most patients dead from hemorrhagic shock. However, based on current evidence supporting its efficacy in these complications, we believe that the infusion of albumin for cirrhosis with GIB is favorable to improve the in-hospital prognosis in those with high-risk for non-bleeding death [16].

In this study, the pre-RBC infusion was positively correlated with the occurrence of rebleeding. It is not surprising that patients with rebleeding present severe bleeding at admission, thus require more RBC transfusion. Although the causal relationship between pre-RBC infusion and rebleeding cannot be determined based on current data, this issue has already been described by many well-designed studies [8, 20, 21]. The limited RBC transfusion reduces the occurrence of rebleeding, the survival benefit was not observed in our study. Its effect on survival was not inconsistent in previous reports probably due to the different enrollment criteria [8, 21–24]. Nevertheless, the restrictive RBC transfusion is a cost-effective strategy as most studies show that less RBC transfusion does not correlate with poor prognosis. Similarly, the casual relationship between pre-ALB and rebleeding illustrated in this study needs to be valided by prospective research.

As a retrospective study, the limitations were inevitable. First, the time of albumin infusion before rebleeding was not evaluated. Normally, the albumin is not recommended in the initial resuscitation of hemorrhagic shock. Whether albumin should be applied in the resuscitation stage or after hemostasis achieved needs to be explored. Second, the average mimum albumin value and dose of albumin used in our study were low compared with previous study [16]. It resulted from the conventional view of albumin effect on GIB, as well as its high cost. Therefore, the necessarity of albumin infusion in patients without hypoproteinemia and the related dosage remains to be clarified. Thirdly, the 12-day follow-up period was shorter than those described in prior studies. As the rebleeding usually occurs within the first 2 weeks, we believe our analysis was still persuasive for the evaluation of the rebleeding event [8]. As its effect on survival, a longer follow-up is required. Lastly, apart from the variables we have included in the analysis, another important confounder is the difference of patient preference in the treatment decision. For example, those patients could afford more albumin were likely to accept endoscopic and radiological interventions. However, the effect of albumin infusion in cirrhosis with GIB has not been systemically studied. Our study would provide a reference for future studies in this subject. In summary, it is speculated that patients of C class would benefit from albumin infusion. Details regarding the previous use of albumin, treatment regimen also needs to be considered in the future study with a longer follow-up period.

## Conclusions

The present study provides the first evidence supporting the application of albumin in the management of cirrhotic patients admitted for acute GIB. Albumin infusion was associated with a lower risk of in-hospital rebleeding in pateints with hypoalbuminemia, Moreover, in Child-Pugh C class, albumin transfusion might be correlated with a decreased number of in-hospital deaths.

## Data Availability

The datasets used and/or analyzed during the current study are available from the corresponding author on reasonable request.

## Abbreviations

GIB: Gastrointestinal bleeding
SBP: Spontaneous bacterial peritonitis
HRS: Hepatorenal syndrome
HCC: Hepatocellular carcinoma
PVT: Portal vein thrombosis
RBC: Red blood cell
ALB: Albumin
